# Identifying Features of Bodily Expression As Indicators of Emotional Experience during Multimedia Learning

**DOI:** 10.3389/fpsyg.2017.01303

**Published:** 2017-07-27

**Authors:** Valentin Riemer, Julian Frommel, Georg Layher, Heiko Neumann, Claudia Schrader

**Affiliations:** ^1^Institute of Psychology and Education, Ulm University Ulm, Germany; ^2^Institute of Media Informatics, Ulm University Ulm, Germany; ^3^Institute of Neural Information Processing, Ulm University Ulm, Germany

**Keywords:** emotion recognition, body posture, body movement, serious games, multimedia learning, game play, generalized estimating equations

## Abstract

The importance of emotions experienced by learners during their interaction with multimedia learning systems, such as serious games, underscores the need to identify sources of information that allow the recognition of learners’ emotional experience without interrupting the learning process. Bodily expression is gaining in attention as one of these sources of information. However, to date, the question of how bodily expression can convey different emotions has largely been addressed in research relying on acted emotion displays. Following a more contextualized approach, the present study aims to identify features of bodily expression (i.e., posture and activity of the upper body and the head) that relate to genuine emotional experience during interaction with a serious game. In a multimethod approach, 70 undergraduates played a serious game relating to financial education while their bodily expression was captured using an off-the-shelf depth-image sensor (Microsoft Kinect). In addition, self-reports of experienced enjoyment, boredom, and frustration were collected repeatedly during gameplay, to address the dynamic changes in emotions occurring in educational tasks. Results showed that, firstly, the intensities of all emotions indeed changed significantly over the course of the game. Secondly, by using generalized estimating equations, distinct features of bodily expression could be identified as significant indicators for each emotion under investigation. A participant keeping their head more turned to the right was positively related to frustration being experienced, whereas keeping their head more turned to the left was positively related to enjoyment. Furthermore, having their upper body positioned more closely to the gaming screen was also positively related to frustration. Finally, increased activity of a participant’s head emerged as a significant indicator of boredom being experienced. These results confirm the value of bodily expression as an indicator of emotional experience in multimedia learning systems. Furthermore, the findings may guide developers of emotion recognition procedures by focusing on the identified features of bodily expression.

## Introduction

In recent years, there has been a growing interest in addressing learners’ emotions during learning with various multimedia learning systems, such as intelligent learning environments, animations, simulations, or serious games, in order to optimize the learning quality (see D’Mello, 2013, for an overview). In this context, “emotions” can be broadly defined as relatively short affective episodes (Rosenberg, 1998) that are initiated by appraisals of particular situations, followed by specific responses (Mauss and Robinson, 2009). The emotional responses involve subjective feelings, physiological changes, and behavior (e.g., Ekman and Friesen, 1967; Wallbott and Scherer, 1986; Frijda, 1988; Scherer, 2005; Larsen et al., 2010).

This recent interest in emotions has been stimulated not only by the realization that diverse design aspects of multimedia learning systems trigger a broad variety of emotions (e.g., Kapoor et al., 2001; Mota and Picard, 2003; Azevedo and Strain, 2011; D’Mello and Graesser, 2012a), but also that learners’ emotional experiences influence learning and its predictors. It is known, for example, that positive emotions such as enjoyment can induce intrinsic motivation (e.g., Pekrun et al., 2002, 2004, 2010). Further, they preserve cognitive resources, direct the attention, and facilitate the application of metacognitive strategies (e.g., Pekrun et al., 2002, 2004, 2010; Meinhardt and Pekrun, 2003). Moreover, positive emotions expedite creative problem solving and more flexible thinking (see Isen, 2001). In contrast, negative emotions such as boredom and frustration–at least in a prolonged manner–might impede learning through diminishing motivation and deep information processing as well as cognitive engagement (e.g., Meinhardt and Pekrun, 2003; Linnenbrink and Pintrich, 2004; Pekrun et al., 2010).

Thus, acknowledging learners’ emotional experience during learning with multimedia learning systems can provide designers with useful feedback that enables an optimization of the interaction between a system and the learners in order to improve learning. As a prerequisite for such an enhancement, emotions experienced by learners need to be documented continuously without interruption of the learning process. To achieve this, previous approaches have sought to recognize emotions from physiological changes and behavior, rather than by using subjective ratings. Examples include the assessments of heart rate and heart rate variability (e.g., Cowley et al., 2013), electrical brain activity (e.g., Heraz and Frasson, 2007), skin conductance (e.g., Picard, 1998), and facial expression (e.g., Kapoor et al., 2007; Azevedo and Strain, 2011; Wiklund et al., 2015). However, the present study focuses on another behavior variable that has not, to date, been studied extensively as a source for emotion recognition in multimedia learning systems: *bodily expression*. Although precise definitions vary, “bodily expression” is generally identified as a variety of posture and activity features of single body parts, such as the arms, the upper body, or the head, as well as of the whole body (e.g., Wallbott, 1998).

The social function of bodily expression is seen to be in communicating emotions to others (Ekman and Friesen, 1967; Darwin, 1872; Wallbott and Scherer, 1986; Frijda, 1988; Scherer, 2001; De Gelder, 2006). More specifically, bodily expression is thought to provide information about an individual’s inclination to engage in a particular action, such as a tendency to approach or withdraw from a situation (Frijda, 1988; Scherer, 2001; De Gelder, 2006). The links between bodily expression, emotions, and action tendency are also implied by studies investigating the role of cerebral asymmetry in emotion expression (e.g., Davidson et al., 1990; Davidson, 1992; Schiff and Bassel, 1996). The left cerebral hemisphere processes approach-related emotions such as happiness (Davidson et al., 1990), and also is related to approach-oriented bodily expressions (Schiff and Bassel, 1996), which include, for example, an expanded body posture or stretched arms (Frijda, 1988; Scherer, 2001). In contrast, the right cerebral hemisphere processes emotions that involve a withdrawal tendency, such as sadness (Davidson et al., 1990), and is associated with withdrawal-oriented bodily expressions (Schiff and Bassel, 1996). Examples of withdrawal-oriented bodily expressions include turning the head or slumping the body (Frijda, 1988; Scherer, 2001).

Questions of whether and which specific features of bodily expression actually drive the recognition of emotions has been investigated in the fields of human emotion perception (e.g., de Meijer, 1989; Wallbott, 1998; Roether et al., 2009; Gross et al., 2010; Dael et al., 2012) and affective computing (e.g., Castellano et al., 2007; Glowinski et al., 2011; Kleinsmith et al., 2011; Gunes et al., 2015). A widely applied research approach is to instruct actors to display several specific emotions in a standing position (e.g., de Meijer, 1989; Wallbott, 1998; Castellano et al., 2007; Gross et al., 2010; Dael et al., 2012), while sitting at a desk (e.g., Gunes et al., 2015), or while walking (Roether et al., 2009). The identification of specific features is either done by using human observers (e.g., Wallbott, 1998; Dael et al., 2012; Gunes et al., 2015) or with a computerized recognition system (e.g., Castellano et al., 2007). Joy and happiness, for example, have been found to be connected with the openness of the whole body, such as an extended upper-body posture (Gunes et al., 2015), laterally stretched and opened arms (de Meijer, 1989; Gunes et al., 2015), and an upward bent head (Wallbott, 1998; Dael et al., 2012). In contrast, a slumped posture of the upper body, closed arms, or keeping the arms at the sides of the body are associated with negative emotions, such as fear, sadness, anger, or anxiety (de Meijer, 1989; Wallbott, 1998; Gunes and Piccardi, 2005; Gunes et al., 2015). Further, Dael et al. (2012) found that moving the whole body forward was used by actors to communicate anger. In contrast, moving backward was associated with fear in a study by de Meijer (1989), and increased shifts in any direction were associated with boredom in Gunes et al. (2015).

As it is argued that the relationship between bodily expression and emotions can vary depending on the situational context (Kleinsmith and Bianchi-Berthouze, 2013), another line of research has investigated how bodily expression relates to genuine emotional experience in actual situations. In the context of multimedia systems, the main focus of the present study, only a few researchers have attempted to investigate the relation between bodily expression and emotional experience in multimedia learning systems (e.g., Mota and Picard, 2003; Woolf et al., 2009; Grafsgaard et al., 2012) and while gaming (e.g., van den Hoogen et al., 2008; Kleinsmith et al., 2011; Witchel et al., 2016). In a study by Grafsgaard et al. (2012), for example, posture and activity features of the upper body and the head were recorded with a depth-image sensor while participants were engaged in a computer-mediated tutoring lesson. Additionally, self-reports of experienced frustration were collected at the end of each lesson. The authors reported that an upper-body posture that was positioned closer toward the computer screen was related to self-reported frustration. Moreover, an increased overall activity of the body (i.e., upper body and head) was also associated with frustration.

The relationship between upper body activity and emotional experience has also been investigated by van den Hoogen et al. (2008), who asked their participants to play a first-person shooter game at a desktop computer. In addition, the participants provided retrospective self-reports not only about their level of frustration, but about the broader range of emotions they had experienced during the game. In line with the findings of Grafsgaard et al. (2012), a positive correlation between frustration and upper body activity was found. In contrast, though, these results showed that enjoyment but also boredom co-occurred with decreased amounts of upper body activity. Regarding head posture and activity, Woolf et al. (2009) analyzed bodily expression and emotion indicators (i.e., valence and arousal) of students using a tutoring software. The authors reported that keeping the head straight was associated with positive valence and lower arousal, whereas turning the head to the side was related to negative valence and higher arousal. In addition, a high amount of head activity was related to negative valence and high arousal. The latter result supports findings relating increased activity to frustration (van den Hoogen et al., 2008; Grafsgaard et al., 2012), an emotion of negative valence and high arousal. A more detailed view of the role of head activity is presented in a study undertaken by Witchel et al. (2016). Comparing the quantity of head movements in differently engaging interactive tasks (i.e., a “boring” reading task vs. an “interesting” reading task vs. playing a shooting game on a desktop computer), the authors found that the participants showed the highest amount of head activity during the boring reading task. This result appears to parallel the findings of Gunes et al. (2015), who reported that increased upper body activity was associated with boredom.

All of the reported results point to the potential of bodily expression for emotion recognition in multimedia learning systems. However, given the limited number of studies addressing genuine emotional experience, its relationship with specific features of bodily expression is far from understood. Hence, in the next section, the contribution of the present study to this particular area of knowledge is delineated.

### The Present Study

Although promising, the results of existing studies on the relationship between bodily expression and genuine emotional experience underscore the need for additional research. Prior reported results are scattered over a few studies that either have used observer ratings to infer emotional experience of individuals (Woolf et al., 2009; Kleinsmith et al., 2011) or assessed emotional experience at single instances in retrospect (van den Hoogen et al., 2008; Grafsgaard et al., 2012). While the former approach makes it difficult to draw conclusions about genuine emotional experience, the latter impedes the consideration of dynamic changes during educational tasks (see D’Mello and Graesser, 2012b). However, previous research has indicated that different emotions show different patterns of change during interaction with a multimedia learning system, and that these patterns of change (and persistence) have distinct impacts on learning (D’Mello and Graesser, 2011, 2012b).

Accordingly, the present study first aims to detect significant changes in emotional experience as well as bodily expression over the course of learning with a multimedia learning system. Based on these data, the second and major aim of this study is to identify features of body posture and body activity that relate to genuine emotional experience, and thereby provide a basis from which to continuously recognize emotions. Thus, the study’s research questions concern (a) whether and how emotions and bodily expression change over the time of learning in a multimedia learning system (i.e., a serious game), and (b) whether and which specific posture and activity features are related to single emotions relevant for learning–namely, enjoyment, boredom, and frustration.

To answer these research questions, a serious game that aimed to enhance the practical money skills of individuals was chosen for use in the present study. The gameplay context was applied because serious games provide interactive and engaging learning experiences that can elicit a wide range of emotions, such as enjoyment, boredom, and frustration, to varying degrees (Garris et al., 2002; D’Mello, 2013). In general, enjoyment in games is connected to design features pertaining to challenge, competition, companionship, exploration, fantasy, and fidelity (Quick et al., 2012). For example, players experience enjoyment when their skills match the game’s challenge as determined by the difficulty level (van Lankveld et al., 2010), when they compete against others (Plass et al., 2013), and when they cooperate with others during gameplay (Plass et al., 2013). Conversely, boredom in games usually occurs when the players’ skills exceed the difficulty of a game (van Lankveld et al., 2010), which results in an exaggerated amount of perceived control (Schrader and Nett, submitted). In addition, boredom can arise in games that require little action from the players (Vorderer et al., 2003). Finally, frustration occurs when the players lack the skills or knowledge to overcome a challenging or competitive situation (Gilleade and Dix, 2004; van Lankveld et al., 2010). Moreover, frustration can also result from a lack in the game’s usability, such as a delayed or no response from the game’s input device (Miller and Mandryk, 2016).

Therefore, to address dynamic changes in emotions during learning with the game, emotional experience was assessed via self-reports at multiple occasions throughout the gaming task. To detect bodily expression, an off-the-shelf depth-image sensor (i.e., Microsoft Kinect for Windows v2) was used, which allowed non-invasive and continuous data collection. Previous research in clinical contexts has shown that Kinect provides reliable and accurate measures of body posture and activity (e.g., Galna et al., 2014; Mobini et al., 2015). For the present setting, only posture and activity features of the upper body and the head were considered. This was because the participants had to operate the mouse and keyboard during gameplay, and, therefore, their arms generally exhibited instrumental movements and postures related to controlling the game rather than to emotional experience (see also Witchel et al., 2016).

By taking a comprehensive approach including a diverse set of emotions as well as features of bodily expression, this study will add to the understanding of the relationship between bodily expression and genuine emotional experience. Moreover, the findings will help developers to focus on the most relevant features of bodily expression as sources for emotion recognition procedures.

## Materials and Methods

### Participants

The sample consisted of 70 right-handed undergraduate students (46 female and 24 male) with a mean age of 22.29 years (*SD* = 2.84). The participants were students in the disciplines of psychology (57.10%), science, technology, engineering, and mathematics (32.80%), medicine (4.30%), and economics (5.80%). All students were enrolled at a German university and were recruited via e-mail invitations and notes posted on campus. For compensation, students could choose to receive either course credit or a payment of 15 euros. This study was carried out in accordance with the recommendations of the ethical committee of Ulm University with written informed consent from all subjects. The participants gave their written informed consent in accordance with the Declaration of Helsinki and with the ethical committee of the authors’ institution.

### Stimulus

**FIGURE 1:**
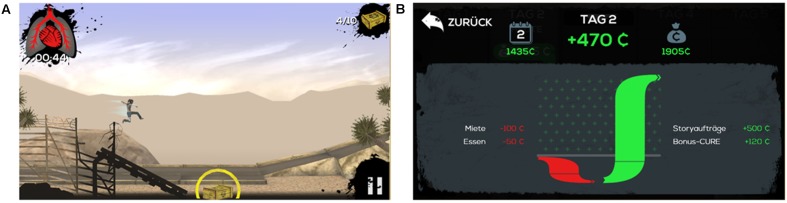
Example screenshots of *Cure Runners*: **(A)** mission, **(B)** decision and reflection phase.

The platform game *Cure Runners* (Three Coins, 2013) was used in this study. The game aims to enhance the practical money skills of juveniles and young adults. It consists of five game sections, each comprising several missions, and decision and reflection phases (see **Figure 1**). The missions are the actual platform levels that need to be accomplished in order to progress from one game section to the next and, ultimately, finish the game. In the missions, the player has to guide an avatar over the platforms and overcome obstacles. Additionally, the player has to collect a number of specific mission items (e.g., boxes with supply goods) in order to accomplish the missions. The challenge for the player is to reach the end of a mission level within a certain amount of time and without letting the avatar fall from platforms. Upon accomplishing the missions, the player earns “Cure,” the game’s currency. The cure can be spent in the decision and reflection phases, which are the game’s phases between the missions. During these phases, the player can monitor his or her current financial state by viewing specific statistics and balance sheet screens. The player also has to decide whether to spend the earned cure on basic needs (e.g., housing and nourishment), gameplay aids (e.g., additional time for mission sequences), or on items without any function in the game (e.g., alternative equipment styles). Additionally, the player can choose between different payment arrangements (e.g., paying immediately, on credit, or by installment) and has to deal with the consequences of his or her decisions (e.g., becoming overindebted).

### Instruments

#### Self-Reports of Emotional Experience

To collect self-reports of emotional experience during playing *Cure Runners*, on-screen questionnaires were administered to each participant after each of the game’s five sections. Each time, participants had to indicate the extent to which they had experienced *enjoyment*, *boredom*, and *frustration* during the previous game section on a seven-point Likert scale ranging from 1 (*very little*) to 7 (*very strong*).

#### Depth-Image Data of Bodily Expression

To collect the features of bodily expression during gameplay, Microsoft Kinect for Windows v2 (Kinect) was used. The system comprises a 1080p color camera and a 512 × 424 pixel time-of-flight light-independent depth image sensor.

In addition, the Kinect for Windows Software Development Kit 2.0 (Microsoft, 2014) was used to interface with the Kinect sensor and its skeletal tracking software, which generates frames of data at a frequency of approximately 30 Hz. Furthermore, an application that stored data on the hard drive whenever the Software Development Kit provided new data was developed and implemented. Using this application, two kinds of data were logged that were of interest for this study: (1) the position of one skeletal joint representing the upper body (i.e., the spine at shoulder height) and (2) the rotation of the head.

**FIGURE 2:**
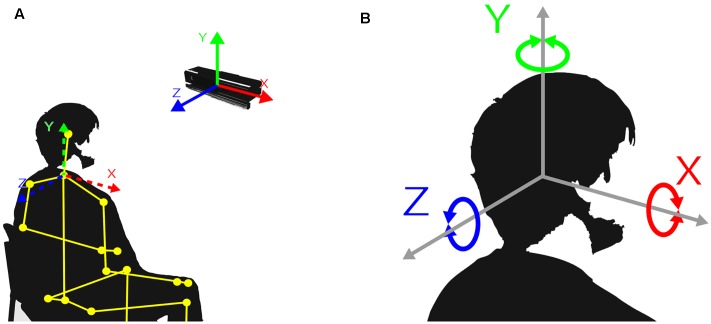
Kinect coordinates: **(A)** body joint positions in relation to the coordinate system of the Kinect; **(B)** head rotation around the axes of the coordinate system of the Kinect.

For the upper body joint, the lateral (left–right, *x*-coordinate), vertical (up–down, *y*-coordinate), and horizontal (forward–backward, *z*-coordinate) positions were stored in relation to the sensor’s camera space (see **Figure 2A**). For the head rotation, the pitch (raised–lowered, *x*-coordinate), yaw (turned to the left–right, *y*-coordinate), and roll (tilted to the left–right, *z*-coordinate) were logged (see **Figure 2B**).

### Procedure

**FIGURE 3:**
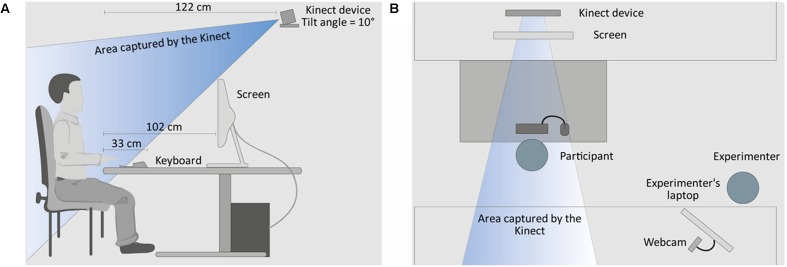
Experimental setup: **(A)** profile view showing the position of the participants in relation to the gaming screen and the Kinect device; **(B)** top view showing the position of the participants in relation to the experimenter.

The study took place in a computer lab on campus. After a brief introduction, the participants completed a questionnaire that ascertained demographic information as well as additional variables that were not the subject of the present study. Subsequently, the participants received a short manual containing gameplay information about *Cure Runners*. Afterward, they were instructed to adopt a relaxed sitting position and face the screen straight ahead. In order to track the posture and activity features of the upper body as well as of the head, the Kinect device was placed above the gaming screen, at a distance of about 120 cm from the participants, and facing downward at an angle of 10° (see **Figure 3A**).

Next, a baseline task was administered in order to record the individual sitting positions. Here, the participants had to perform a series of activities, including pressing either one of four keys (W, A, S, or D) of the keyboard or the left mouse button to answer on-screen prompts. The same inputs were also used later as controls for the gameplay. The participants did not receive any feedback on the correctness of their inputs. After 60 s, the baseline task ended and the game started immediately. The participants played until they completed the fifth section of *Cure Runners*. During the baseline task and over the course of the game, participants’ bodily expressions were recorded by the Kinect device. Between the game sections, the emotion questionnaires were administered to the participants using an event-based Wizard-of-Oz setting. The experimenter watched the participants’ gaming screens through a hidden webcam connected to a laptop (see **Figure 3B**) and remotely started the questionnaires whenever the participants had completed a game section. Furthermore, whenever the participants wanted to pause the game, they had to push an on-screen button, which called up a “Stop” symbol screen overlay. The mean duration of the complete procedure was 86.12 min (*SD* = 15.75). Upon completion, the participants were debriefed and received their compensation of choice.

### Data Processing

In order to differentiate the baseline task, the five game sections, the on-screen questionnaires, and possible game pauses, the gaming sessions were screen-captured by using OBS Multiplatform (Open Broadcaster Software, 2015). Based on the screen recordings, specific events were identified that indicated the on- and offsets of relevant (i.e., the baseline task and each of the five game sections) and non-relevant phases (i.e., on-screen questionnaires and game pauses) of the gaming sessions. An offline template-matching procedure was applied that identified the events based on predefined characteristic image patches. After the relevant phases were identified, data from the Kinect device and from the template-matching procedure were synchronized, allowing the temporal assignment of bodily expression to the particular phases of the gaming sessions. For that purpose, Kinect data as well as on- and offsets of the game phases were annotated with time stamps. Based on these time stamps, both data sources were merged using a Python script, assigning a Kinect data point recorded between an on- and an offset to the respective game phase. The non-relevant phases were excluded from further processing.

Because the Kinect device was tilted (see **Figure 3A**), the *y*- and *z*-axes of the upper body joint were not independent–that is, joint displacements on the *z*-axis caused displacements on the *y*-axis, and vice versa (see **Figure 2A**). However, no relevant changes were expected to occur in the upper body joint on the *y*-axis (i.e., vertical displacements), and, therefore, the *y*-coordinate was excluded from further processing. In contrast to the upper body joint, the head rotation remained unaffected by the positioning of the Kinect device; thus, all coordinates were included in the analyses.

The posture and activity for each coordinate were calculated as means over the recorded frames of each of the five game sections. For each coordinate *i* in each game section *j*, the mean posture was calculated as:

$${Mean\,Posture}_{{Coord}_{i}{Sect}_{j}}=\frac{\Sigma_{k=1}^{m}\left(x_{ik}\right)}{m}$$

with *x* being the value of coordinate *i* in the *k*th frame of the Kinect recording in game section *j*, and *m* being the total number of frames in game section *j*.

Accordingly, the mean activity in each game section was calculated as:

$${Mean\,\;Activity}_{{Coord}_{i}{Sect}_{j}}=\frac{\Sigma_{k=1}^{m}|x_{ik}-x_{ik-1}|}{m}$$

The mean posture and activity coordinates for the baseline task were calculated analogously to those for the game sections. In order to correct for individual differences in the sitting positions, the mean posture and activity for each of the five game sections were centered at the respective coordinates obtained for the baseline task. Thus, the final posture and activity features were calculated as:

$${Posture}_{{Coord}_{i}{Sect}_{j}}={Mean\,\;Posture}_{{Coord}_{i}{Sect}_{j}}-{Mean\,\;Posture}_{{Coord}_{i}Baseline}$$

$${Activity}_{{Coord}_{i}{Sect}_{j}}={Mean\,\;Activity}_{{Coord}_{i}{Sect}_{j}}-{Mean\,\;Activity}_{{Coord}_{i}Baseline}$$

**Table 1** provides an overview over the posture and activity features of bodily expression used in this study and the meanings of the correspondent coordinate values.

**Table 1:** Descriptions of bodily expression features and meanings of correspondent coordinate values.

Bodily expression feature	Meaning of coordinate values
Upper-Body Posture *X*	Keeping the upper body displaced to the *left* (negative values) or to the *right* (positive values) compared to the baseline position when facing the Kinect sensor
Upper-Body Posture *Z*	Keeping the upper body displaced *closer to* (lower values) or *further away from* (higher values) the Kinect sensor compared to the baseline position
Head Posture *X*	Keeping the head more *raised* (positive values) or *lowered* (negative values) compared to the baseline position
Head Posture *Y*	Keeping the head more *turned* to the *left* (positive values) or to the *right* (negative values) compared to the baseline position
Head Posture *Z*	Keeping the head more *tilted* to the *left shoulder* (positive values) or to the *right shoulder* (negative values) compared to the baseline position
Upper-Body Activity *X*	Lower vs. higher total amount of upper body movement to the *left and right* compared to the baseline activity when facing the Kinect sensor
Upper-Body Activity *Z*	Lower vs. higher total amount of upper body movement *closer to and further away from* the Kinect sensor compared to the baseline activity
Head Activity *X*	Lower vs. higher total amount of *lowering and raising* the head compared to the baseline activity
Head Activity *Y*	Lower vs. higher total amount of *turning* the head to the *left and right* compared to the baseline activity
Head Activity *Z*	Lower vs. higher total amount of *tilting* the head to the *left and right shoulder* compared to the baseline activity


### Statistical Analysis

To answer the questions of whether and how emotions change during learning with a serious game, self-reported enjoyment, boredom, and frustration as well as features of bodily expression were analyzed for within-subject differences between the five game sections. Based on the results from Kolmogorov–Smirnov tests for normality and the inspections of skewness, Friedman’s analysis of variance (ANOVA) was used for self-reported emotions and the activity features of bodily expression. Parametric repeated measures ANOVA was only used for the posture features of bodily expression. Because of technical failure (e.g., dropouts in the Kinect recordings), complete data over all game sections was only available in relation to a limited number of participants. Thus, the sample sizes for the tests of within-subject differences were reduced to *n* = 55 for emotions and to *n* = 32 for features of bodily expression.

To answer the questions of whether and which specific posture and activity features relate to the emotions, generalized estimating equations (GEEs), as introduced by Liang and Zeger (1986), were applied. GEEs allow regression analyses of several time-dependent variables by accounting for the within-subject correlation of repeatedly measured variables (Ballinger, 2004). Thus, the value of bodily expression as an indicator for emotional experience could be analyzed in consideration of between- as well as within-subject variability. As already mentioned, data on bodily expression features showed missing values in several game sections. However, GEE can handle incomplete data when values are missing completely at random (Ballinger, 2004), which was confirmed through application of the test proposed by Little (1988) [χ^2^ = 704.46 (727), *p* = 0.719]. Therefore, GEE models could be obtained by using the full sample size of *n* = 70.

In GEEs, the distribution of the dependent variable (i.e., the self-reported emotions) and the structure of the within-subject correlations for the repeated measures of the dependent variable must be specified. For the present study, the inverse Gaussian distribution was specified, because the self-reported emotions were not normally distributed but exposed positively skewed distributions. Following a suggestion by Ballinger (2004), the autoregressive within-subject correlation structure was specified for all emotions, because the data represented a time-series structure.

In order to identify the significant features of bodily expression indicating self-reported emotions, a stepwise procedure was applied for each emotion separately. In the first step, an initial GEE model was established with all features of bodily expression included as independent variables. In the next step, the non-significant features were excluded and new GEE models were calculated. The fits of the emerging GEE models were compared, based on the corrected quasi-likelihood under the independence model criterion proposed by Pan (2001). The new GEE model was only processed further when its fit exceeded the fit of the preceding model. This procedure was repeated until, for each emotion, the best fitting model, which comprised a subset of significant features of bodily expression, was identified.

## Results

### Descriptive Statistics

**Table 2** provides the descriptive statistics for self-reported emotional experience and bodily expression for each game section. For better readability, the raw values of the posture and activity features were multiplied by 100.

**Table 2:** Means and standard deviations for self-reported emotional experience and bodily expression over the five game sections.

	Game section 1^a,b^	Game section 2^c,d^	Game section 3^e,f^	Game section 4^g,h^	Game section 5^i,j^
Variable	*M* (*SD*)	*M* (*SD*)	*M* (*SD*)	*M* (*SD*)	*M* (*SD*)
Emotions					
Enjoyment	4.23 (1.28)	3.81 (1.60)	3.48 (1.61)	3.73 (1.69)	3.33 (1.69)
Boredom	1.56 (0.90)	1.67 (1.28)	1.70 (1.42)	1.72 (1.40)	1.76 (1.32)
Frustration	2.69 (1.41)	3.23 (1.73)	3.44 (1.79)	2.78 (1.77)	3.15 (1.77)
Bodily Expression Features					
Upper-Body Posture *X*	0.67 (1.77)	0.13 (1.47)	0.03 (2.28)	0.20 (2.04)	0.31 (1.67)
Upper-Body Posture *Z*	-0.18 (4.42)	1.34 (6.58)	1.56 (7.92)	1.55 (8.71)	1.64 (7.83)
Head Posture *X*	0.10 (3.22)	0.26 (4.30)	-0.67 (4.90)	0.25 (5.26)	0.32 (4.66)
Head Posture *Y*	-2.86 (3.49)	-2.69 (3.29)	-2.98 (4.03)	-3.27 (4.12)	-3.30 (4.07)
Head Posture *Z*	-0.43 (2.32)	-0.79 (2.55)	-0.62 (2.52)	-1.41 (2.51)	-1.68 (2.57)
Upper-Body Activity *X*	1.10 (3.17)	0.18 (0.80)	0.56 (2.20)	0.03 (0.19)	0.12 (0.54)
Upper-Body Activity *Z*	0.41 (1.23)	0.04 (0.24)	0.16 (0.58)	0.02 (0.01)	0.07 (0.28)
Head Activity *X*	0.30 (0.69)	0.16 (0.46)	0.36 (0.92)	0.43 (0.92)	0.42 (0.76)
Head Activity *Y*	0.25 (0.60)	0.26 (0.72)	0.49 (0.89)	0.46 (0.92)	0.48 (0.72)
Head Activity *Z*	0.05 (0.35)	0.04 (0.35)	0.09 (0.48)	0.14 (0.52)	0.18 (0.33)


*Values of bodily expression features were multiplied by 100 for better readability. For emotions: ^a^*n* = 70, ^c^*n* = 70, ^e^*n* = 64, ^g^*n* = 60, ^i^*n* = 55; for bodily expression features: ^b^*n* = 53, ^d^*n* = 57, ^f^*n* = 49, ^h^*n* = 51, ^j^*n* = 45.*

### Changes in Emotional Experience and Bodily Expression during Gameplay

**FIGURE 4:**
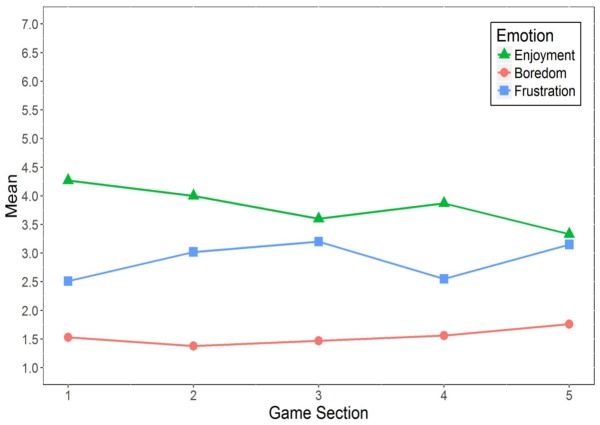
Changes in emotional experience during playing *Cure Runners. n* = 55.

The changes in the self-reported emotions over the course of the game are depicted in **Figure 4**.

For *enjoyment*, participants in general reported medium levels and the values decreased over the course of the game, except between game sections 3 and 4. A Friedman’s ANOVA revealed significant differences in participants’ enjoyment between the game sections [$\chi_{F}^{2}\left(4\right)$ = 25.47, *p* < 0.001, *n* = 55].

For *boredom*, participants reported comparably low values throughout the game. However, values increased over the five game sections and showed significant differences [$\chi_{F}^{2}\left(4\right)$ = 10.64, *p* = 0.031, *n* = 55].

Finally, *frustration* appeared to generally increase during participants’ playing of *Cure Runners*, except between game sections 3 and 4. Self-reported frustration also differed significantly over the five game sections [$\chi_{F}^{2}\left(4\right)$ = 24.85, *p* < 0.001, *n* = 55].

**FIGURE 5:**
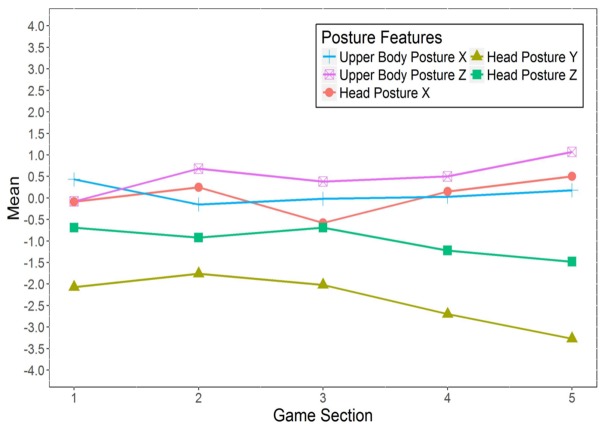
Changes in the posture features of bodily expression during playing *Cure Runners*. Means multiplied by 100. *n* = 32.

For the *upper-body posture* features (see **Figure 5**), the values of Upper-Body Posture *X* are just above zero (i.e., the individual baseline position) throughout the game. Thus, the participants in general adopted an upper-body posture slightly displaced to the right, compared to their individual baseline postures. In addition, given the values of Upper-Body Posture *Z*, the participants adopted an upper-body posture further away from the screen, compared to their individual baseline postures, except for during the first game section. However, no significant differences for the means of Upper-Body Postures *X* and *Z* were found between the five game sections.

Regarding the *head posture* features (see **Figure 5**), in most of the game sections, the participants kept their heads more raised than in their baseline postures, as indicated by the largely positive values of Head Posture *X*. In the third game section, however, the participants kept their head more lowered, compared to their baseline. In addition, the participants kept their heads more turned toward the right (negative values of Head Posture *Y*), and more tilted toward their right shoulder (negative values of Head Posture *Z*), compared to their respective baseline head postures. The turning and tilting of the head to the right appeared to increase over the course of the game. Repeated measures ANOVAs further revealed significant differences between the game sections for Head Posture *Y* [*V* = 0.41, *F*(4,28) = 4.84, *p* = 0.004, $\eta_{p}^{2}$ = 0.41] and Head Posture Z [*V* = 0.38, *F*(4,28) = 4.20, *p* = 0.009, $\eta_{p}^{2}$ = 0.38].

**FIGURE 6:**
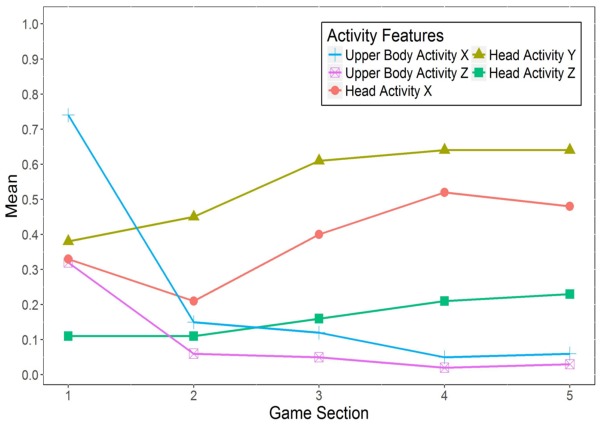
Changes in the activity features of bodily expression during playing *Cure Runners*. Means multiplied by 100. *n* = 32.

For the *activity features* of the upper body and the head (see **Figure 6**), the positive values indicate that participants generally exhibited more activity while playing *Cure Runners* than during the baseline task. In addition, upper body activity appeared to decrease while head activity seemed to increase over the course of the game. However, no significant differences between the five game sections were found for any of the activity features.

### Indicators of Bodily Expression for Emotional Experience

As reported in Section “Changes in Emotional Experience and Bodily Expression during Gameplay,” the means of the self-reported emotions across all participants varied significantly between the game sections. To control for possible effects of measurement wave on emotions, the game sections were included as an additional independent variable in the initial GEE models. The parameter estimates of the final GEE models are given in **Table 3** for each self-reported emotion separately. In order to assess the relative strengths of the parameter estimates, the scales for each variable were transformed to range from 0 to 1 by using the proportion of maximum scaling method (Little, 2013; Moeller, 2015).

**Table 3:** Estimates of game section and bodily expression features indicating self-reported emotional experience.

Emotion	Independent variable	*B*	*SE B*	χ^2^(1)	*p*
Enjoyment					
	Game Section	-1.05	0.25	17.46	<0.001
	Head Posture *Y*	1.82	0.89	4.13	0.042
	Head Posture *Z*	-1.75	1.00	3.09	0.079
Boredom					
	Head Activity *Z*	1.18	0.41	8.26	0.004
Frustration					
	Upper-Body Posture *Z*	-1.02	0.51	4.10	0.043
	Head Posture *Y*	-3.28	0.70	21.70	<0.001


*Variables were transformed to scales ranging from 0 to 1 prior to the analyses.*

#### Enjoyment

The effect of game sections on self-reported enjoyment across participants (see Changes in Emotional Experience and Bodily Expression during Gameplay) is also reflected in the final GEE model. As the negative sign of *B* indicates, enjoyment decreased over the course of the game. However, the *y*-coordinate of the head posture emerged as the strongest indicator for self-reported enjoyment. The positive sign of *B* indicates that, the more participants kept their heads turned toward the left (in relation to their individual baseline postures), the higher their self-reported enjoyment was while playing *Cure Runners*. In addition, as the negative *B* for the *z*-coordinate shows, keeping the head more tilted toward the right shoulder (in relation to the baseline) also indicated higher experience of enjoyment. However, this feature was only marginally significant.

#### Boredom

In the final GEE model, the activity of the head around the *z*-axis emerged as the only significant feature indicating self-reported boredom during gameplay. The more participants exhibited tilting movements of the head (relative to their baseline activity), the more they reported being bored during gameplay, compared to participants who showed fewer head tilting movements.

#### Frustration

The posture of the head rotated around the *y*-axis emerged as the strongest indicator for frustration experienced during playing *Cure Runners*. The negative value of *B* in **Table 3** indicates that, the more participants kept their heads turned toward the right (in relation to their individual baseline postures), the higher their self-reported frustration was. Additionally, the posture of the upper body along the *z*-axis was also a significant indicator of frustration. Relative to the baseline, the closer the individuals kept their upper bodies toward the game screen, the higher their self-reported frustration.

## Discussion

The present study set out to investigate the changes in and the relationship between bodily expression and genuine emotional experience during learning with a serious game. Accordingly, the dynamic changes in emotional experience over the course of gameplay were addressed by using repeated self-reports of emotions. Furthermore, bodily expression during gameplay was recorded and specific features of bodily expression that are most relevant as indicators of distinct emotions were identified.

In respect of the first research question regarding changes in emotions over the course of playing *Cure Runners*, self-reported emotional experience varied significantly throughout the game (see **Figure 4**). Specifically, enjoyment generally decreased over time, whereas frustration generally increased. An explanation for this may be that the majority of the participants found the game hard to play, as recorded in verbal comments after the gaming sessions. Therefore, they may have experienced a discrepancy between their gaming skills and the challenge of the game, which caused their experience of frustration to increase (see Gilleade and Dix, 2004). Boredom was experienced only to a small degree, as indicated by the mean scores (see **Table 2**). However, it increased significantly over time, which may have been a consequence of the prolonged experience of frustration (D’Mello and Graesser, 2012b).

In contrast to the self-reported emotions, significant changes in bodily expression over the course of serious gaming were found only for 2 out of 10 features; namely, the postures of keeping the head turned and tilted. Most noticeably, throughout the game, an increase in keeping the head turned to the right, compared to participants’ individual baseline positions, was found (see **Figure 5**). Together with the changes in enjoyment and frustration (see **Figure 4**), this result already points to a link between head turning and emotional experience.

Concerning the second research question regarding the relationship between specific features of bodily expression and emotional experience, posture and activity features of the upper body and the head were shown to indicate self-reported emotional experience during the playing of a serious game. Moreover, different emotions were either indicated by different features or, in the case of enjoyment and frustration, shared a feature with different signs. More specifically, adopting a posture of the upper body positioned more closely to the gaming screen (i.e., Upper-Body Posture *Z*) indicated self-reported frustration. This result is in line with the finding of Grafsgaard et al. (2012), who reported the same relation between upper-body posture and frustration. Given that the participants were sitting at a desk, the adoption of a position closer to the screen may have indicated a slumped body posture (i.e., leaning on the desk; see also Witchel et al., 2016). Such a posture has been considered to communicate withdrawal-related emotions (Frijda, 1988; Wallbott, 1998), and frustration can be accompanied by withdrawal when individuals are unable to cope with the frustrating event (Harmon-Jones et al., 2003). Thus, participants adopting a posture that was closer to the screen possibly indicated a withdrawal tendency in the face of insurmountable frustration in the present study.

The posture feature of the head turned around the vertical axis (i.e., Head Posture *Y*) emerged as the strongest indicator for two emotions. Keeping the head turned to the left indicated self-reported enjoyment, whereas keeping the head turned to the right was a sign of frustration. This result is partly in line with findings relating a head posture turned to the right to acted emotion displays of withdrawal-related emotions (Gunes et al., 2015). Moreover, the present finding aligns with results from neuropsychological studies, relating left-sided muscle contractions to withdrawal tendencies and right-sided contractions to approach tendencies (Schiff and Bassel, 1996). In the present study, a left-sided muscle contraction in the neck, which is responsible for keeping the head turned to the right (Gatterman, 2012), was related to frustration, whereas the opposite applied for enjoyment. In addition, a right-sided muscle contraction also causes the tilting of the head to the right shoulder (Gatterman, 2012), which emerged as a marginally significant indicator for enjoyment. Thus, paralleling the finding regarding upper-body posture, keeping the head turned to the right might have indicated the withdrawal tendency accompanying the experience of frustration. In contrast, keeping the head turned to the left (and tilted to the right shoulder) may have expressed the approach tendency accompanying enjoyment.

An alternative interpretation is provided by considering that the participants kept their heads generally more turned to the right, as indicated by the negative means of Head Posture *Y* in **Table 2**. Therefore, even comparably high individual values of Head Posture *Y* may still have denoted a head posture turned to the right. Consequently, the positive relation between Head Posture *Y* and enjoyment (see **Table 3**) may actually mean that keeping the head more straight (i.e., less turned to the right) indicated enjoyment. This interpretation is in line with findings relating a straight head posture to positive emotions and a turned head posture to negative emotions (Woolf et al., 2009).

The amount of head tilting activity emerged as a significant positive indicator for boredom, which conforms to the findings of Witchel et al. (2016). In addition, the result resembles findings relating increased bodily activity to low attention (Farrace-Di Zinno et al., 2001) and disengagement (Woolf et al., 2009). As a possible explanation, some authors (see Garger, 1990) have argued that increased bodily activity serves the activation of the neural system in order to refocus attention on a lengthy task. Thus, an increased head tilting activity in the present study may have reflected an attempt to cope with the experience of boredom during gameplay.

The present study has some notable limitations. Due to the specified research approach, combinations of features were not analyzed. For example, how Head Posture *Y* and Upper-Body Posture *Z* interact as indicators of frustration was not addressed. Moreover, due to the application of a regression procedure, the present results have to be interpreted as relations. Therefore, no information can be provided about “cut-off” values or intervals of bodily expression features that may indicate whether or not an emotion is experienced. In addition, although the results showed that different emotions were indicated by different features of bodily expression, the discriminative power of the identified features was not assessed directly. This remains an open task for future studies.

Another limitation concerns the narrow set of parameters (i.e., mean of posture and overall activity) we used as features of bodily expression. Other possible parameters include the range of posture and activity as well as the speed, acceleration, and direction of activity. In the future, an increased effort is needed to analyze and compare these additional parameters to achieve more reliable bodily expression indicators of emotional experience.

Regarding the measure of emotional experience, the use of self-reports can be criticized, because it can interrupt the gameplay experience (Pagulayan et al., 2003). In order to overcome this issue without relying on observer ratings, retrospective self-reports that are aided by letting the participants view their own gaming sessions can be applied in the future (e.g., D’Mello and Graesser, 2011). However, for the present study, this procedure was considered to be too onerous for the participants because of the length of the gaming sessions. Another limitation of using self-reports of emotional experience concerns their susceptibility to individual differences in alexithymia. Individuals high in alexithymia are assumed to be less aware of their own emotional states, although they react to emotional stimuli (Lane et al., 1997). Thus, the reliability of emotion self-reports can be diminished for high alexithymia individuals.

Further, only a limited number of discrete emotions was investigated in the present study. While the emotions were chosen for their proven relevance in learning activities (e.g., Pekrun et al., 2002, 2004, 2010), additional emotions and affective-cognitive states, such as confusion (e.g., D’Mello and Graesser, 2012b), can impact learning in multimedia learning systems and need to be considered in future studies.

Finally, the Kinect had dropouts during the recordings, possibly because it was not always able to fit a skeleton to the participants as their lower body was obstructed due to the study’s setup. Although this did not pose a problem for the analysis, it diminishes the reliability for practical applications. Nonetheless, the Kinect device offers a low-priced and easily implemented way to gain exhaustive data on bodily expression for the purpose of research.

Despite these limitations, the present study provides substantial support for the applicability of bodily expression in recognizing genuine emotional experiences. In contrast to previous works that assessed single instances of emotions in retrospect (e.g., van den Hoogen et al., 2008; Grafsgaard et al., 2012) or used observer ratings (e.g., Woolf et al., 2009; Kleinsmith et al., 2011), the present study related bodily expression to repeatedly collected self-reports of emotions during a task. Thus, within-subject changes in the emotional experience were accounted for, and, consequently, the ecological validity of the present results could be increased. In addition, by using GEE models, the features of bodily expression that showed the highest relative importance compared to other features for a given emotion could be identified. This analytic approach helped to avoid ambiguities, such as for bodily activity, which has been previously related to boredom (Witchel et al., 2016) but also to frustration (van den Hoogen et al., 2008; Grafsgaard et al., 2012).

The findings of this study not only add to our understanding of the relationship between bodily expression and emotional experience, but also, by identifying specific features of bodily expression as relevant indicators for emotional experience, the results can inform developers of automatic emotion recognition systems. Moreover, the present findings may apply to other contexts in which a desktop computer is operated, such as entertainment games. The practical application of the present findings will be the subject of future works, aiming to further advance emotion recognition for multimedia learning systems.

## Author Contributions

All authors contributed to the conception of the work. VR, JF, and CS designed and conducted the study. JF wrote the software codes for the Wizard-of-Oz setup, the logging of the Kinect data, and the synchronization of the Kinect data with the game phases. GL wrote the software code to identify the game phases from the on-screen recordings. VR conducted the statistical analyses, VR and CS wrote the first draft, and all authors revised the final manuscript.

## Conflict of Interest Statement

The authors declare that the research was conducted in the absence of any commercial or financial relationships that could be construed as a potential conflict of interest.
